# AI-assisted early screening, diagnosis, and intervention for autism in young children

**DOI:** 10.3389/fpsyt.2025.1513809

**Published:** 2025-04-14

**Authors:** Sijun Zhang

**Affiliations:** Institute of Educational Sciences, Hunan University, Changsha, China

**Keywords:** autism, artificial intelligence, early screening, early diagnosis, early intervention, young children, machine learning

## Abstract

Autism is a serious threat to an individual’s physical and mental health. Early screening, diagnosis, and intervention can effectively reduce the level of deficits in individuals with autism. However, traditional methods of screening, diagnosis, and intervention rely on the professionalism of psychiatrists and require a great deal of time and effort, resulting in a large proportion of individuals with autism being diagnosed after the age of 6. Artificial intelligence (AI) combined with machine learning is being used to improve the efficiency of early screening, diagnosis, and intervention of autism in young children. This review aims to summarize AI-assisted methods for early screening, diagnosis, and intervention of autism in young children (infants, toddlers, and preschoolers). To achieve early screening and diagnosis of autism in young children, AI methods have built predictive models to improve the automation of early behavioral diagnosis, analyzed brain imaging and genetic data to break the age barrier for diagnosis, and established intelligent screening systems for early mass screening. For early intervention of autism in young children, AI methods built intelligent education systems to optimize the teaching and learning environment and provide individualized interventions, constructed intelligent monitoring systems for dynamic tracking, and created intelligent support systems to provide continuous support and meet the diverse needs of young children with autism. As AI continues to develop, further research is needed to build a large and shared database on autism, to generalize and migrate the effects of AI interventions, and to improve the appearance and performance of AI-powered robots, to reduce failure rates and costs of AI technologies.

## 1 Introduction

Autism spectrum disorder is a neurodevelopmental disorder characterized by deficits in social communication and the presence of restricted interests and repetitive behaviors (1). Autism poses a serious threat to the physical and mental health of individuals and causes significant impairment in social interaction, learning, work, and family life. The causes of autism are complex and varied, including genetic, environmental, and maternal factors. Although the disorder has some genetic causes, due to large individual differences in molecular genetic indicators such as susceptibility genes, clinical diagnosis is currently based on observable and measurable behavioral indicators, for example, parents of autistic patients are asked to complete a multi-item interview scale accompanied by a psychiatrist, who then evaluates the results and makes a diagnosis (2). The current diagnostic process is highly dependent on the professionalism of the psychiatrist, and many test items make the testing process lengthy.

Early intervention can effectively reduce the degree of deficits in social attention, language, and intelligence as well as the overall severity of symptoms in individuals with autism (3). Although symptoms of autism usually appear before the age of 2 years, large-scale early screening is not currently practiced in many regions due to a lack of human resources and diagnostic efficiency. A large proportion of individuals with autism are diagnosed after the age of 6, thus missing the optimal period for behavioral treatment and severely affecting their cognitive development and future quality of life (4). Therefore, how to improve the efficiency of screening and diagnosis of autism in young children (infants, toddlers, and preschoolers) to achieve early screening, diagnosis, and intervention has become an urgent issue. The development of accurate biomarkers is an effective way to enable early screening, diagnosis, and intervention, and artificial intelligence is an effective tool for pattern recognition and correlation analysis. Data processing methods using artificial intelligence can reduce the number of items required to diagnose autism, while shortening the diagnostic process and automating it through model training to maximize the efficiency of clinical diagnosis (2, 5). The application of artificial intelligence to behavioral interventions for autism has also attracted the attention of psychiatrists, for example, monitoring the emotions of an autistic infant using intelligent devices is expected to compensate for the shortcomings of traditional interventions in terms of resources and effectiveness (6). In recent years, researchers have realized the great development space of AI in the early screening, diagnosis, and intervention of autism in young children, and have conducted a lot of research.

Some reviews summarized the research work on autism screening using AI methods (7–9), others reviewed diagnosis using AI for autism (9–14), and some researchers reviewed treatment using AI for autism (14, 15), but these reviews covered the research work for all ages, and these reviews largely ignored research in infants. Given the urgent need for screening, diagnosis, and intervention of autism in infants, toddlers, and preschoolers, this review summarizes AI-assisted methods for early screening, diagnosis, and intervention of autism in young children.

## 2 Methodology

A search of the following databases was completed on 15 September 2024: PubMed, PsychInfo, Ovid Medline, Cumulative Index to Nursing and Allied Health Literature, Web of Science, and Scopus. Search terms included “AI autism infant”, “AI autism toddler”, “AI autism preschool”, “AI autism preschooler”, “AI autism children”, “AI ASD children”, “AI ASD infant”, “AI ASD toddler”, “AI ASD preschool”. In addition, review articles on AI-assisted screening, diagnosis, or intervention for autism were read to ensure that as many articles as possible on AI-assisted early screening, diagnosis, or intervention for autism were included in this review. Search results were limited to English language studies published in the last 15 years.

Studies were included if they met the following criteria: (1) studies showed how AI aided the screening, diagnosis, or intervention of autism, (2) studies used infants, toddlers, or preschoolers as participants, (3) studies were published as journal articles or conference proceedings. Studies that did not include a quantitative evaluation were excluded.

The title and abstract of all papers identified using the above search terms were independently reviewed by two assessors (SZ, JY) according to the inclusion and exclusion criteria. Full-text articles of potentially eligible studies were then reviewed independently by two assessors and categorized for inclusion or exclusion, with clear reasons given for any exclusions. Disputes regarding inclusion were resolved by discussion among all authors (SZ, JY, ZT), Cohen’s K coefficient (0.97) for inter-author agreement indicates perfect agreement among authors.

**Figure 1:**
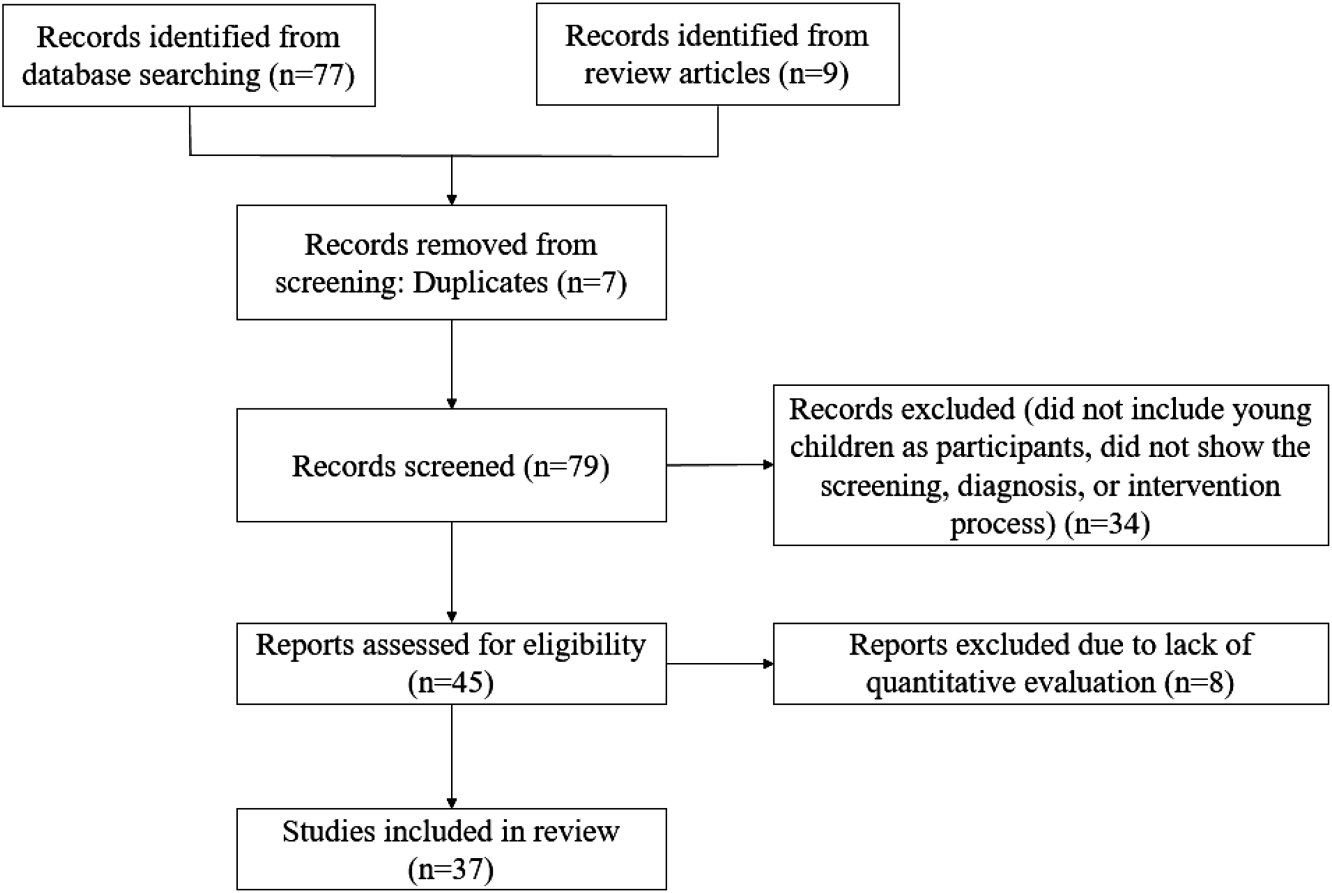
PRISMA 2020 flow diagram of the article selection process.

The initial search identified 86 publications (see **Figure 1**). After removing 7 duplicates, 34 publications were excluded because they did not meet the inclusion criteria (i.e. they did not include young children as participants or did not show the screening, diagnosis, or intervention process). Of the 45 papers that met the criteria for full-text review, a further 8 were subsequently excluded due to lack of quantitative evaluation. The remaining 37 papers were included in the review of AI-assisted early screening, diagnosis, and intervention for autism in young children.

## 3 AI-assisted early screening and diagnosis of autism in young children

Traditional screening and diagnosis use diagnostic tools based on DSM-5, such as Autism Diagnostic Observation Schedule and Autism Diagnostic Interview-Revised Edition. In recent years, as the genetic etiology of autism has been studied in depth, researchers have attempted to use neuroimaging and genetic testing techniques to distinguish individuals with autism from typically developing individuals (16, 17). Although the reliability of these screening and diagnostic methods is high, the screening and diagnostic process is time consuming and does not allow for early screening, diagnosis, and intervention of autism in young children. AI-assisted methods use behavioral and radiomic data to build predictive models and then diagnose based on new test data to improve diagnostic efficiency (8), the diagnostic process requires expert supervision and testing of the predictive effectiveness of the models.

### 3.1 Building predictive models to improve automation of early behavioral diagnosis

Traditional methods of early screening for children with autism require psychiatrists to use behavioral diagnostic tools to identify symptoms of autism in a child’s development based on parental descriptions and clinical observations, and to make subsequent referral recommendations (18). On the other hand, artificial intelligence methods such as machine learning, can automate the autism diagnostic process by learning data categories from a large number of historical autistic samples and control samples and building predictive models that then classify new input samples (19). For example, Wall et al. used machine learning to build an alternating decision tree model based on the Autism Diagnostic Interview-Revised Edition, with input data from the Autism Genetic Research Exchange repository (a repository whose data came from Boston). The alternating decision tree model contained only 7 items, which was 93.3% less than the Autism Diagnostic Interview-Revised Edition, and the output showed that the model effectively diagnosed autistic children aged 13 to 48 months with an accuracy of 99.97% (20).

At the same time, the high heterogeneity among autistic individuals and the comorbidity with other disorders make diagnosis difficult. As an early application of artificial intelligence, expert systems have been developed to simulate the knowledge and reasoning processes of domain experts (21). For example, Adarraga et al. simulated the diagnostic process of experts by constructing a three-level tree diagram of variables through an expert system that abstracted variables from input data. This system was able to successfully distinguish autistic infants from infants with schizophrenia, intellectual disability, and other disorders, but the process required the user to interact with the system in real time through utterances, and the user had to monitor the diagnostic process, the degree of automation is low (5). Bertoncelli et al. used machine learning to build a predictive model to identify whether preschoolers in Nice, France with cerebral palsy also have autism. The predictive model analyzed data on etiology, diagnosis, spasticity, epilepsy, clinical history, communication skills, behaviors, intellectual disability, motor skills, and eating and drinking skills, and found that motor skills, feeding skills, type of spasticity, intellectual disability, and communication disorders in preschoolers with cerebral palsy were associated with autism. The model had an accuracy of 73%, a sensitivity of 79%, and a specificity of 72% and could be used for the early diagnosis of autism in preschoolers with cerebral palsy (22).

Deep learning involves multiple artificial neural networks and associated machine learning algorithms, a deep learning model can extract the characteristics, features, and relationships it needs to make accurate predictions from raw, unstructured data (23). Researchers compared deep learning models generated by different algorithms to classify individuals suspected of having autism and found that deep learning models based on deep neural networks and convolutional neural networks were more effective, outperforming early machine learning algorithms such as support vector machines and Bayes nets in terms of accuracy, sensitivity, and specificity (24–26). With the advantages of freedom, convenience, and efficiency, deep learning has been widely used in many fields such as speech recognition, computer vision, and natural language processing. Deep learning also offers new possibilities for automated diagnosis of young children with autism (26).

### 3.2 Analyzing brain imaging and genetic data to break the age barrier for diagnosis

Using brain imaging data to determine whether brain structure and function are abnormal is another effective way to diagnose autism at an early stage. Radiomics, which combines imaging techniques (e.g., CT, MRI, PET) with artificial intelligence models to identify and diagnose complex pathological patterns in the brain, is a rapidly developing field of medical research with great potential for disorder diagnosis (27). Traditional imaging methods require radiologists to segment the acquired 2D or 3D images to select regions of interest, which is a time-consuming and costly process. Artificial intelligence models help to automate and semi-automate the segmentation process, quickly extracting hundreds and thousands of features from the region of interest, analyzing these features to form a preliminary predictive model, and then validating the model using a new dataset. The above process is suitable for complex pattern analysis and diagnosis of psychiatric disorders (28).

In genetic studies, a large number of family studies and twin studies have demonstrated the high heritability of autism, with 10% to 30% of autism being caused by genetic abnormalities (29). In autism, the high heterogeneity of individual genes and risk factors not only increases the difficulty of genetic testing, but also discourages screening for known susceptibility gene status. Machine learning algorithms such as support vector machines, random forest, and logistic regression can be used to prioritize autism mutation genes, prioritize risk gene discovery, minimize false positives, and facilitate early triage of young children with autism (30). Joudar et al. used machine learning methods based on random forest and found two autism genes: MYC Binding Protein 2 and Phosphatase and Tensin Homolog genes (10). In Michigan, Bahado-Singh et al. used machine learning and deep learning methods to perform epigenome-wide association analysis of neonatal leukocyte DNA. They identified 15 methylation genes out of 249 genes, some of which showed epigenetic dysregulation associated with the development of autism. The deep learning method performed particularly well, with a sensitivity of 97.5% and a specificity of 100%, and could effectively improve diagnostic efficiency and facilitate genetic screening for autism in newborns (31). The diagnosis of autism using brain imaging and genetic data can be independent of the level of cognitive development and language ability of young individuals, which is favorable for early screening and diagnosis. The introduction of artificial intelligence methods can prioritize autism risk genes and diagnose through accurate classification of brain imaging data.

### 3.3 Applying eye tracking with deep learning techniques for early diagnosis

Eye movements as a window into behavior, cognition, and decision-making, have served as promising biomarkers in autism (32). Previous findings suggest that individuals with autism have impairments in perceiving social scenes, establishing and maintaining eye contact, and recognizing facial information, which could be objectively measured using eye-tracking technology (33). Eye-tracking assessments generate large-scale temporal and spatial sequence data and multiple visual attention features that could be learned by deep learning algorithms for early diagnosis of autism (34).

Recently, there has been a significant increase in research using eye-tracking combined with deep learning to classify young children with autism. In these studies, social stimuli and a combination of social and non-social stimuli were the main types of eye tracking stimuli used, with non-social stimuli used only once (35). For deep learning algorithms, Jaradat et al. used deep neural network (DNN) (36), Cheekaty et al. and Ahmed et al. used convolutional neural network-recurrent neural network (CNN-RNN) (37, 38), deep CNN was used in two studies (39, 40), convolutional neural network -artificial neural network (CNN-ANN) was used by Benabderrahmane et al. (41). The diagnostic accuracy of these studies ranged from 80.5% to 98.33%. In a systematic review, Wei et al. found that the diagnostic accuracy was highest in preschoolers compared with school-aged children, adolescents and adults (42).

### 3.4 Establishing intelligent screening systems for early mass screening

Improving the diagnostic efficiency of autism requires both reducing the diagnostic time and expanding the scope of early autism screening from hospitals to communities. Shahamiri et al. developed an autism screening system using convolutional neural networks and integrated it into a mobile application. An autism dataset of 6075 samples from infants, toddlers, preschoolers, adolescents, and adults in New Zealand was used to build the model, which enabled autism screening for individuals younger than 36 months. The autism screening system worked well for young children with autism, with an accuracy of 97.95%, a sensitivity of 95.53% and a specificity of 98.63% (43). This screening system was able to meet the needs of autistic individuals, family members, carers, psychiatrists, and teachers, and could also be used in community health services to expand autism screening and improve the efficiency of clinical diagnosis.

Sohl et al. integrated an artificial intelligence-based diagnostic device into an existing autism screening tool to create a diagnostic model for 18-to 72-month-old children (children from rural and suburban areas of the United States) at risk of developmental delay. The AI model accepted three different behavioral inputs: a short questionnaire about the child’s behavior completed by the caregiver via smartphone, two short home videos uploaded by the caregiver to a video analytics website, and a questionnaire completed by a psychiatrist and uploaded to a healthcare provider’s website. The model decided whether an individual had autism based on these inputs, 1/3 of the sample received a timely and accurate diagnostic assessment, and the model also minimized false negatives (44). Another study compared the consistency of the output of Sohl et al.’s device with expert diagnoses for children with developmental delays between 18 and 72 months from urban areas of California. The results showed that the output of the device had a positive predictive value of 80.8% and a negative predictive value of 98.3%, with a sensitivity of 98.4% and a specificity of 78.9% (45). This AI-based diagnostic device can be used not only for offline healthcare services, but also for online telemedicine management to serve potential autistic individuals in remote or rural areas. Even when access to community healthcare or clinical diagnosis is significantly reduced (e.g., public health emergencies such as COVID-19), the device can be used as an important adjunct screening system.

Robotics also facilitates intelligent screening and assessment of autism. Based on the imitation deficits of autistic infants, Wijayasinghe et al. attempted to assess the imitation skills of autistic infants using robotics. The robot made upper body movements that the infants had to imitate, and the infants’ final movements were captured by a motion capture system and compared with the expected movements to determine the severity of the infants’ imitation deficits (46). Dehkordi et al. used an animal-based robot as a screening tool for preschoolers with autism, the robot assessed the behavioral differences between autistic preschoolers and normal preschoolers exhibited during interaction with the robot, and the screening accuracy was as high as 90% (47). Robotics has a positive role to play in the early screening and diagnosis of autism.

## 4 AI-assisted early intervention for autism in young children

Within the neurodiversity perspective, individuals with autism are not individuals with a set of deficits, but rather individuals who have difficulty adapting to the mainstream environment due to neurodevelopmental variability (48). Educational and social interventions are key factors in improving the social adaptability of individuals with autism. Artificial intelligence can help young children with autism perform basic social interactions and interpersonal communication, and improve their ability to recognize and manage emotions. AI can also help young children with autism to enjoy equal rights to education and work. In the community, AI can help young children with autism make better use of all kinds of facilities and reduce their difficulties in social life.

### 4.1 Applying intelligent education systems to optimize the teaching and learning environment and provide individualized interventions

Autistic individuals have different thinking, learning, and behavioral responses than typically developing individuals, their over- or under-responsiveness to sensory input and abnormal arousal to environmental stimuli can affect their attention and learning performance (49). Artificial intelligence can help improve the cognitive and learning abilities of young children with autism. Sula et al. used the SmartBox device, a device that incorporates artificial intelligence technology, to improve vocabulary and math skills in autistic preschoolers. The device was equipped with multiple receptors and was connected to physical objects in the environment via an RS232C port. The device could sense the hand and body movements of the preschoolers, enhance the preschoolers’ attention through lights, smells, and sounds, and also interact with the preschoolers to improve their concentration and language skills (50). In addition, the use of intelligent robots in the training of young children with autism, such as social interaction and motor skills, helps to improve the training effect. Compared to traditional training, intelligent robots have significant advantages in improving young children’s motor skills and physical coordination, improving stereotypic behaviors and emotional states, and promoting the development of social interaction. Although imitating other humans is the best way to learn social behavior, human social behavior is too subtle and complex. Improving the social and communication skills of young children with autism by using robots as a medium can reduce the interference of irrelevant information (51).

Artificial intelligence can assist with real-time classroom analysis to help teachers develop appropriate teaching strategies. Lampos et al. recorded five months of observations of teachers’ classroom instruction with seven autistic children in a kindergarten, and then examined teachers’ teaching styles and communication strategies, as well as the children’s emotional states and responses. Lampos et al. used machine learning methods based on logistic regression with elastic net regularization, random forest, and composite Gaussian process, to predict which teaching style and communication strategy would maximize autistic children’s responses. Overall, all three machine learning methods predicted higher than random levels of accuracy, with the composite Gaussian process having the highest accuracy; the results demonstrated the potential use of machine learning in kindergarten classroom practice (52). Singh et al. applied deep learning algorithms to the design of a social robot with powerful computer vision, speech recognition, and emotional expression capabilities. The social robot was able to teach arithmetic and daily living skills to autistic preschoolers by presenting the teaching steps more effectively and engagingly, correctly determining the learning effects, and providing real-time feedback (53). Machine learning can improve the convenience of learning while providing personalized educational intervention for autistic preschoolers.

Learning and improving social behavior in young children with autism often takes place in environments that are equipped with technological tools. Artificial intelligence can both communicate and interact with young children with autism and simulate teacher-student interactions in the classroom. AI can analyze the effectiveness of different intervention methods, as well as the characteristics of individuals with autism. AI can effectively predict which communication strategies young children with autism will respond positively to, providing teachers with appropriate communication strategies to improve the level of response of young children with autism in the classroom.

### 4.2 Applying intelligent monitoring systems for dynamic tracking

Individuals with autism have difficulties with social interaction in six ways: 1) difficulty with normal social interactions as well as dealing with conflict situations, 2) difficulty with perceiving, recognizing, and producing facial expressions, 3) difficulty with maintaining eye contact and making eye contact, 4) inability to gaze at objects of interest or to pay direct attention to the target object, 5) an inability to express their needs and feelings through typical gestures or language, and 6) inability to adapt to normal environmental changes and respond to things in a normal way (54). To address these difficulties, researchers have attempted to improve the social interactions of young children with autism through computer vision, social robotics, and virtual reality.

Computer vision technology is designed to help machines “see” and “imagine” the world, thereby enabling the automatic recognition of facial expressions and the design of computer-aided systems based on them (55). The systematic review by De Belen et al. confirmed that computer-aided systems could help individuals with autism to improve their emotional understanding and recognition of facial expressions, and to communicate effectively with others. The effectiveness of teaching with computer-aided systems was comparable to that with humans (56). In addition, computer-aided intervention courses were more interesting than traditional intervention methods, the instructions in the intervention courses were more acceptable and enjoyable to young children with autism, and the intervention process maintained higher accuracy and less variability over multiple repetitions (57). Moreover, as computerized systems are automated, they can better serve remote areas and extend the reach of the intervention. Based on the intervention principle of applied behavioral analysis and machine learning, Ananth et al. built a base model to track and monitor the facial attentional state of young children with autism. The base model extracted eye movement information, performed visual analysis through convolutional neural networks, judged whether to make eye contact, provided appropriate audio feedback based on the individual’s gaze duration, and reinforced the individual’s positive performance. The base model effectively tested the possibility of applying artificial intelligence algorithms to quantum visual tracking and quantum visual processing, which, combined with the intervention principle of applied behavioral analysis, could improve and strengthen eye contact in young children with autism (58). Bekele et al. developed a robotic system that incorporated intelligent tracking technology. The system consisted of a humanoid robot and several wall-mounted cameras, the cameras were responsible for tracking the individual’s head movements in real time to improve the robot’s field of view, and the robot provided appropriate audio feedback based on the individual’s gaze duration to promote joint attention in young children with autism (59).

Mobile applications, immersive virtual reality, and social robots can monitor the emotions and behavior of young children with autism in real time and combine games and training to help young children with autism learn essential social skills. These methods are applicable in a wide range of settings and can be integrated into medical training programs as well as daily training programs for young children with autism, as young children are more receptive to these methods.

### 4.3 Applying intelligent support systems to provide continuous assistance and meet diverse needs

Improving the quality of life for young children with autism requires building smart facilities on a larger scale, as well as community support and government policies. Researchers proposed combining IoT devices with artificial intelligence technology to build smart cities. In a smart city, intelligent technologies would be integrated into various facilities, such as roads, restaurants, transportation, offices, homes, and educational institutions. Through these technologies, individuals with autism could receive assistance with their daily activities (60).

Stable community intervention services depend on a functioning and organized health care delivery system. Due to the social skills deficits of individuals with autism, they are likely to be in a poor position when some sudden public health events (e.g., COVID-19) occur. In response to this status quo, Banna et al. designed an artificial intelligence support system to meet the needs of children aged 3 to 12 with autism in specific situations. The support system consisted of a monitoring system and an emotion recognition system. The monitoring system consisted of an intelligent wristband, an interactive monitor, and a camera attached to the monitor; these devices were connected to a mobile application installed on a mobile phone so that caregivers could continuously monitor the status of autistic children. The emotion recognition system recognized the facial expressions of autistic children with 78.56% accuracy. During COVID-19, the support system showed autistic children the importance of hand washing and wearing masks through virtual games, monitored their movement and health status, optimized their learning styles based on their emotional state, and provided companionship and educational functions when the number of caregivers was insufficient (61). Artificial intelligence combined with IoT technologies can help to monitor and intervene with young children with autism on a wider scale, reducing young children’s dependence on caregivers and psychiatrists with the help of robots, mobile apps, and smart monitoring systems, etc. Artificial intelligence combined with IoT technologies will allow young children with autism to live in a continuous and controlled learning environment, acquiring the necessary skills for better development.

## 5 A look at the future of artificial intelligence for young children with autism

### 5.1 Building a large and shared autism database

The development of artificial intelligence has brought new ideas to autism screening, diagnosis and intervention. The advantage over traditional methods is that AI can process and analyze large amounts of data, making the screening, diagnosis and rehabilitation of young children with autism easier and more effective. However, the use of large datasets inevitably raises concerns about the privacy and security of young children with autism. Some researchers proposed the use of autism spectrum disorder testing apps to collect data on individuals with autism of all ages, including characteristics such as age, gender, jaundice at birth, and ethnicity (62). Other researchers suggested that sensors in smartwatches could be used to collect behavioral data from young children with autism and send it to the cloud for analysis (63). However, there was no detailed explanation of how user privacy would be protected in these studies. Numerous researchers postulated that AI developers should put serious first consideration on ethics and observe regulatory standards of AI technologies for health care. This pits several aspects that touch on privacy and data protection, informed consent issues, and general issues of accountability when AI technologies are deployed into clinical settings (64). In addition, the data used to build autism diagnostic models were mostly from different datasets or different versions of the same dataset. To address the problem of unbalanced category labelling (e.g., fewer females in the dataset), researchers integrated datasets from various sources to improve the predictive model, and data limitations increased the difficulty of testing the reliability of the model. Moreover, most studies about AI-assisted early screening, diagnosis, and intervention for autism in young children have been conducted in a few developed countries (USA, France, Germany, New Zealand, etc.), the majority of participants have been from urban areas, the generalizability of the results is unknown, and more research is needed from other populations.

The diversity of data is the basis for providing personalized interventions for young children with autism, so there is a need to build large autism databases with broad applicability and full category labelling. Meanwhile, clinicians or psychiatrists can collect autism diagnostic data in real time using portable devices, such as tablets, personal digital assistants, or even smartphones. This process requires the government to take effective administrative and technological measures to protect the privacy and security of the participants in autism databases. Specific measures include clarifying the recording and storage of information, setting data access and transfer rights, ensuring secure data storage and anonymizing data collection. On the basis of information security, more autistic children and their families will be encouraged to participate voluntarily in order to enrich the database and build a more accurate mathematical model of the behavior of autistic children, which will support the diagnosis and treatment of autism.

### 5.2 Migrating and extending the effects of AI interventions

Artificial intelligence is seen by technology enthusiasts as “a new way to improve the accuracy and effectiveness of autism diagnosis” (65). Young children with autism are also more receptive to information provided by high-tech products such as AI than traditional educational devices (66). However, while AI products may improve the skills of young children with autism, they may also cause them to become addicted to such products, thus reducing their interaction and communication with the outside world (67). Future research needs to work in two directions. On the one hand, when using AI in interventions for young children with autism, learning activities can be designed to be more realistic. On the other hand, small and portable smart devices or smart apps can be designed to provide continuous assistance and support to young children with autism after they leave the training site, thus facilitating the transfer of acquired skills to everyday life.

Currently, AI intervention for young children with autism only focuses on social interaction, language and cognitive development, and stereotyped behavior, but in the future, AI devices will need to make technological breakthroughs to expand the scope of intervention from the core obstacles to other related areas. Specifically, the AI will need to enable collaborative medical and educational training to improve social interaction, mimicry, turn-taking behavior, joint attention, collaborative play skills, communication and sharing skills, and interest in learning, while enabling medical diagnostic treatments through a powerful platform.

### 5.3 Improving the appearance and performance of AI-powered robots

With the development of artificial intelligence, robotics is becoming increasingly integrated into special education. A large number of studies have demonstrated the effectiveness of robots as a medium for screening, diagnosis, and intervention for autism in young children (51). Robots are ideal for simplifying social behaviors to facilitate learning in young children with autism and offer many advantages during autism interventions. Compared to human interventions, robot-assisted interventions are more controllable, interference from irrelevant information can be minimized, and robots do not get tired and can perform their work continuously and efficiently (68).

Currently, there are three main types of robots used for assisted interventions: machine-like robots, animal-like robots, and humanoid robots (69). The review by Saleh et al. confirmed that young children with autism are more interested in humanoid robots and that the intervention is more effective than with non-humanoid robots (70). In certain interactive environments, young children with autism will show behaviors such as eye contact, imitation, responding to the robot, and even actively approaching the robot (71). However, the current robots used for autism intervention have relatively weak interaction capabilities and cannot make real-time adjustments according to the emotional state of autistic children (72). Most existing robots can only mimic the user’s upper body movements, including head and arm movements, and their flexibility and range of motion are very limited. Considering the balance state of the robot and the use of forward kinematics, in the future, we need to design robots that can mimic the bending and rotation of the legs and waist (73), to increase the degree of free movement of the robot, so that young children with autism can mimic the whole-body movements of the robot.

Some researchers have raised the moral and ethical issues that may arise when robots are designed to look like humans; for example, young children with autism may take humanoid robots as their partners and become attached to them, while robots, if destroyed, may cause psychological harm to young children (74). In addition, it is unclear whether the social and communication skills exhibited by young children with autism during their interactions with robots are durable, and whether these skills can be transferred to young children’s everyday lives. In the future, from an ethical point of view, the relationship between young children with autism and robots, and the role of robots in everyday life, needs to be further clarified to avoid over-reliance on robots and to realize the transition of the intervention effect from human-machine to human-human and human-daily life.

As young children with autism are a diverse and heterogeneous group, it is necessary to design the robot according to their psychological characteristics, interests, and hobbies, to improve the robot’s personalized settings and personalized service capabilities, so as to achieve personalized intervention. In addition, robots are more often used in the intervention of young children with autism using traditional behavioral intervention models, such as combining robotics with applied behavioral analysis, stimulus-response-reinforcement method, and social skills training in laboratory situations, which lack the effect test in real situations. Future research can consider a more ecological behavioral intervention model that takes full advantage of robots and provides real-life situations and good experiences for young children with autism.

### 5.4 Reducing failure rates and costs of AI technologies

Failure rates for AI-assisted screening and diagnosis ranged from 0.03% to 19.5%. The failure rate correlated with the age of the participants, and the accuracy of screening and diagnosis for autism was highest in preschoolers. The low diagnostic accuracy of autism in infants is due to a delay in the development of databases of infants with autism (9); more research is needed in infants, whether using traditional diagnostic methods or AI-assisted methods. In addition, studies combining imaging techniques with AI models had lower failure rates than studies without imaging techniques, suggesting that imaging techniques are essential for AI-assisted diagnosis of autism in infants (75).

Implementation AI for early screening, diagnosis, and intervention of autism in young children may be very costly, beyond the means of most health care institutions, especially those with limited resources. Although AI can potentially reduce health care costs in the long term by being efficient and improving health outcomes, the high level of investment required in infrastructure, training, and integration of AI services could be substantial (76). Most of the studies in this review were conducted in high-resource settings with state-of-the-art imaging equipment and computing infrastructure. However, the implementation of these AI technologies in low- and middle-income countries, where healthcare resources are scarce, faces significant challenges. Research is urgently needed to modify AI technologies for use in such settings, focusing on technologies that require less computational power, can work with limited or lower-quality autistic data, and are cost-effective. The lack of studies in this area exacerbates global inequalities, and addressing this gap is critical to ensuring that AI can benefit young children with autism worldwide, regardless of geographic or economic barriers (77).
